# Cells Dynamically Adapt to Surface Geometry by Remodeling Their Focal Adhesions and Actin Cytoskeleton

**DOI:** 10.3389/fcell.2022.863721

**Published:** 2022-06-03

**Authors:** Aysegul Dede Eren, Amy W. A. Lucassen, Urandelger Tuvshindorj, Roman Truckenmüller, Stefan Giselbrecht, E. Deniz Eren, Mehmet Orhan Tas, Phanikrishna Sudarsanam, Jan de Boer

**Affiliations:** ^1^ Department of Biomedical Engineering, Institute for Complex Molecular Systems, Eindhoven University of Technology, Eindhoven, Netherlands; ^2^ MERLN Institute for Technology Inspired Regenerative Medicine, Maastricht University, Maastricht, Netherlands; ^3^ Laboratory of Physical Chemistry, Department of Chemical Engineering and Chemistry, Eindhoven University of Technology, Eindhoven, Netherlands

**Keywords:** mechanotransduction, focal adhesion, tenocytes, cell shape, stress fibers

## Abstract

Cells probe their environment and adapt their shape accordingly *via* the organization of focal adhesions and the actin cytoskeleton. In an earlier publication, we described the relationship between cell shape and physiology, for example, shape-induced differentiation, metabolism, and proliferation in mesenchymal stem cells and tenocytes. In this study, we investigated how these cells organize their adhesive machinery over time when exposed to microfabricated surfaces of different topographies and adhesive island geometries. We further examined the reciprocal interaction between stress fiber and focal adhesion formation by pharmacological perturbations. Our results confirm the current literature that spatial organization of adhesive sites determines the ability to form focal adhesions and stress fibers. Therefore, cells on roughened surfaces have smaller focal adhesion and fewer stress fibers. Our results further highlight the importance of integrin-mediated adhesion in the adaptive properties of cells and provide clear links to the development of bioactive materials.

## Introduction

“Contact guidance” is a term coined by Paul Weiss in 1945 to describe that nerve cells adapt their shape to geometrical patterns of the substrate on which they grow (Weiss, 1945), be it epithelial hydra *in vivo* (Campbell and Marcum, 1980) or microfabricated topographical surfaces *in vitro* (Bettinger et al., 2006). It illustrates that cell constantly probe their surroundings and adapt their interaction accordingly. Cell shape, adhesion, and actin organization are intricately linked in their relation to the topographical design of the substrate (Robotti et al., 2018; Vasilevich et al., 2020). This is very clear when comparing cells on flat vs. topographical surfaces. Flat surfaces lead to cells with very large focal adhesions and abundant stress fiber formation, and cells have a very high area and are flat (Vasilevich et al., 2020). On topographies, the cell area is typically smaller, cells are higher, have smaller focal adhesion, and fewer stress fibers (Dalby et al., 2004). For instance, Cassidy et al. (2014) reported that mesenchymal stem cells and osteoprogenitors have a spread morphology, higher surface tension, and large focal adhesions (bigger than 8 µM in length) on flat surfaces while grooved substrates, cells were elongated and possessed smaller focal adhesions (1–5 µM in length). Similarly, Baharloo et al. (2005) demonstrated that the epithelial cell area was larger on smooth surfaces and had more and larger focal adhesions compared to roughened surfaces.

This phenomenon can be explained with the cellular tensegrity model that proposes that cells are normally in a pre-stressed state, which is actively created by the actomyosin-based contractile apparatus of cells and coordinated with the cell’s adhesion to the extracellular matrix (ECM) (Wang et al., 2001; Ingber, 2003). Tension can only be created when the surface on which the cells grow permits it. For instance, cells on surfaces with high stiffness allow the creation of high tension and force on focal adhesions without distortion of the matrix, and thus allow larger focal adhesions and more tension in their cytoskeleton (Prager-Khoutorsky et al., 2011). Cells on surfaces with low stiffness have smaller focal adhesions (Prager-Khoutorsky et al., 2011). On rigid substrates, cells display high spreading and large and uniformly distributed focal adhesions compared to softer substrates on which cells possess radially oriented focal adhesions with smaller cell sizes (Pelham and Wang, 1997; Prager-Khoutorsky et al., 2011; Fusco et al., 2015; Abagnale et al., 2017). Similarly, cells have fewer adhesion points on roughened surfaces, and can therefore create less tension *via* smaller focal adhesions (Bershadsky et al., 2006; Thievessen et al., 2013; Legerstee et al., 2019). In turn, cell shape follows the ability of the cells to form focal adhesions. The formation of focal adhesions, actin cytoskeleton, and the forces placed on it is well regulated and determined by the geometry of the substrate.

The cells’ adaptation to substrate geometry has consequences for cell physiology (Von Erlach et al., 2018). The material properties of ECM influence mechanosensitive signaling pathways, which control the expression of genes involved in differentiation, proliferation, and metabolism (Beijer et al., 2019; Vasilevich et al., 2020), (Jansen et al., 2017). We and others have previously investigated the relationship between surface topography and cell physiology in tenocytes (English et al., 2015) and mesenchymal stem cells (McNamara et al., 2010). Tenocytes possess a spindle-shaped morphology in their native tendon ECM but upon *in vitro* culture become spread with a bigger cell area and a lower aspect ratio, as well as an increased number of stress fibers, and they, lose the expression of typical tendon marker genes, such as tenomodulin, scleraxis, and Mohawk (Yao et al., 2006). Rat tenocytes cultured on tendon imprint and microtopographies led to elongated cell morphology, reduced cell area, fewer stress fibers, and higher expression of scleraxis (Vermeulen et al., 2019; Dede Eren et al., 2020). The same was observed in human mesenchymal stem cells, another frequently used cell type in tenogenic differentiation studies (Kishore et al., 2012; Younesi et al., 2014; Dede Eren et al., 2020; Vermeulen et al., 2020). The Rho/ROCK signaling pathway is one of the pathways engaged in the link between adhesion and phenotype. Rho proteins are involved in various biological processes including cell shape and actin cytoskeleton organization (Sit and Manser, 2011), and non-muscle myosin-II is involved in actin–myosin interactions (Vicente-Manzanares et al., 2009). In several studies, the involvement of Rho/ROCK/SRF signaling in the tenocyte phenotype was demonstrated by targeting this pathway with small molecules (Xu et al., 2011; Maharam et al., 2015; Madhurakkat Perikamana et al., 2018).

In our previous reports, we focused particularly on the downstream phenotypic consequences of different topographies on tenocytes (Dede Eren et al., 2021a) and mesenchymal stem cells (Dede Eren et al., 2020; Vermeulen et al., 2020; 2021). We demonstrated that tenocyte marker gene expression is induced on topographic imprints of the tendon collagen bundles (Dede Eren et al., 2021a). Using our TopoChip platform, we recently reported that human mesenchymal stem cells (hMSCs) gene expression and phenotypical responses, such as differentiation, proliferation, and apoptosis, strongly correlate to cell shape (Hulshof et al., 2017), and also found evidence that actin mediated signaling processes play a role based on cell shape (Vermeulen et al., 2020). Therefore, in this study, we aimed to unravel the dynamic interaction between tenocytes and their native environment, and we explored how this ventures out to microfabricated topographies in hMSCs to further understand tenocytes. With the results of this study, to our knowledge for the first time, the effect of native tendon topography on the organization of the focal adhesion and F-actin architecture on tenocytes are reported.

## Materials and Methods

### Surface Fabrication

A detailed description of TopoChip fabrication of the microtopographies can be found elsewhere (Unadkat et al., 2011). In brief, the inverse pattern of topographies in polystyrene (PS) was etched into a silicon wafer, by deep reactive ion etching, generating a silicon master mold. The master was coated with a layer of perfluorodecyltrichlorosilane (FDTS, Sigma-Aldrich) and copied into polydimethylsiloxane (PDMS). The PDMS intermediate (mold) was subsequently copied into OrmoStamp hybrid polymer (micro resist technology GmbH), which served as the working mold for hot embossing the topographies into PS films (Goodfellow). The conditions for the hot embossing were 140°C for 5 min at a pressure of 10 bar, and a demolding temperature of 90°C. Before cell culture, PS films were oxygen plasma-treated for 30 s at 0.8 mbar, 50 sccm O2, and 100 W to enhance cell attachment.

Production of tendon imprint is described elsewhere (Dede Eren et al., 2020). Briefly, porcine tendons were sectioned longitudinally at 300 µM thickness with a cryotome (Leica CM1950). Next, PDMS solution (SYLGARD 184 silicone elastomer base and elastomer curing agent) was prepared in 10:1 ratio and subsequently poured onto the tendon slices and vacuum degassed for 30 min. Next, PDMS was allowed to polymerize for 48 h. Next, the tendon section was peeled off from PDMS, creating a native tendon topography. For the production of the PS tendon imprint, the following construction was clamped: glass slide—PDMS mold—PS sheet—glass slide and was put in the oven at 140°C for 30 min. After cooling down, the PS was removed from the mold and underwent plasma oxygen treatment (30 s, O_2_). All surfaces were sterilized with ethanol for at least 30 min and washed with PBS before cell culture. A flat PMDS mold that does not contain any topography was used to create PS flat surfaces as control groups.

To produce the platform with binary micropatterns, a two-step thiol-ene reaction was performed. Before thiol-ene coupling on the surface of a microscopy glass slide, the glass was activated with oxygen plasma and piranha solution. Then, the vinyl silane was vapor-deposited overnight at 80°C. Next, a two-step thiol-ene reaction was performed on the vinyl-coupled surface. First, 10 mM CGGGRGDS (Chinapeptides) peptide solution containing 5 mg/ml LAP [lithium phenyl-2,4,6-trimethylbenzoylphosphinate (TCI chemicals)] solution was prepared. On the vinyl-modified glass substrate (25 × 25 × 1 mm, width × length × thickness), 3 ul of the RGD thiol solution was dropped, covered with the photomask, and irradiated with UV light for 10 min. The volume of thiol solution for the first thiol-ene reaction is critical to transfer the patterns as a (proximity) gap between mask and glass substrate reduces the resolution of the patterns. After removing the photomask, the samples were washed in ethanol, followed by water in an ultrasonication bath for 10 min, and dried with a nitrogen gun. Then, the second thiol to graft (10 mM of either polyethylene glycol thiol or HAVDI peptide solution containing 5 mg/ml photoinitiator) was dropped onto the pre-patterned surface, covered with a fluorinated quartz slide, and UV-irradiated for another 10 min. Finally, the plate was washed with THF, ethanol, and water in an ultrasonication bath and dried with a nitrogen gun. The samples were stored in 70% ethanol for further use at 4°C.

### Cell Culture

Human mesenchymal stem cells (hMSCs) isolation was conducted, as previously described (Beijer et al., 2019), from the bone marrow of a 74-year old female donor after obtaining written informed consent from the patient. Ethical approval for using the samples was obtained from the ethical advisory board of the Medisch Spectrum Twente, Enschede. All methods were carried out following local and relevant guidelines and regulations. hMSCs were cultured in basic medium [alpha-minimum essential medium (α-MEM), (Gibco)] supplemented with 10% fetal bovine serum (FBS) (Thermo Fisher Scientific) and 1% penicillin/streptomycin. To investigate the effect of surface topography, passage 5 hMSCs were seeded at 5,000 cells/cm^2^ density and cultured for the designated times in a humidified incubator with 5% CO_2_ at 37°C.

Rat tenocytes were isolated from the hind limbs of 23 weeks old Cyp1a2ren strain rats after euthanization, considering their surplus status from the breeding program as previously described (Dede Eren et al., 2020). Upon cell isolation, tenocytes were expanded in a cell culture medium of Dulbecco’s modified Eagle’s medium (DMEM, Sigma-Aldrich) supplemented with 10% FBS and 100 U/ml penicillin/streptomycin. To investigate the effect of surface topography, passage 4 tenocytes were seeded at 5,000 cells/cm^2^ density and cultured for the designated times in a humidified incubator with 5% CO_2_ at 37°C. Bovine chondrocytes were isolated from bovine articular cartilage from the metacarpo/tarsophalangeal joint, provided as a slaughterhouse material, by using an overnight digestion protocol. In brief, the digestion solution contains chondrocyte medium [high-glucose DMEM (Gibco), 10% FBS (Sigma-Aldrich), and 100 U/ml penicillin/streptomycin (Thermo Fisher Scientific)], 0.01% hyaluronidase, and 0.15% collagenase II. Passage 2 chondrocytes were used in this study. HeLa cells (ATTC CRM-CCL-2™, 70012009) were cultivated in high-glucose DMEM with pyruvate (Gibco), 10% FBS (Sigma-Aldrich), and 100 U/ml penicillin/streptomycin (Thermo Fisher Scientific). HeLa cells were used at passage 9. Human monocytes were isolated using CD14 microbeads human (BD, 130-050-201) from peripheral blood mononuclear cells (PBMCs) by following the protocol described in the datasheet. CD14^+^ cells were seeded 10,000 cells/cm^2^ using complete RPMI 1640 medium containing 10% FBS (Sigma-Aldrich), 100 U/ml penicillin/streptomycin. All cells were seeded at 5,000 cells/cm^2^ unless stated otherwise.

### Pharmaceutical Reagents Exposure

hMSCs were seeded on Topo1018 surface topographies at the seeding density of 5,000 cells/cm^2^ in a basic medium supplemented with manganese (II) chloride (MnCl_2_) (Sigma-Aldrich) at a final concentration of 2 mM to activate integrins, paclitaxel (Sigma-Aldrich) at a final concentration of 1 µM to stabilize microtubule arresting, parbendazole (Bio-Connect B.V) at a final concentration of 4 µM to inhibit the microtubule assembly. As paclitaxel and parbendazole were dissolved in DMSO, we added a DMSO control, and its concentration in the final culture media was 4 µM. We also added RGD peptides Gly-Arg-Gly-Asp-Ser-Pro and Gly-Arg-Gly-Asp-Ser-Pro-Lys (Sigma-Aldrich) to block integrin activation, Gly-Arg-Ala-Asp-Ser-Pro (Sigma-Aldrich) as a negative control. Cells were exposed to pharmaceutical reagents for 4 h upon cell seeding.

### Scanning Electron Microscopy

Samples were prepared as previously described (Dede Eren et al., 2020). Briefly, rat tenocytes were cultured as described earlier and fixed in 2.5% glutaraldehyde (Fisher Scientific) at room temperature for 1 hour. Then, they were washed with distilled water three times for 10 min, dehydrated in 25, 50, 75, 90, and 100% ethanol for 15 min each, and incubated in 100% ethanol for additional 15 min. Next, the samples were dried in hexamethyldisilazane (HMDS) (Sigma-Aldrich) for 1 h. Before imaging, samples were coated with 5 nm gold–palladium and imaged using a scanning electron microscope (SEM) (FEI Quanta 3D FEG Dual Beam).

### Immunofluorescent Staining

The samples were prepared as previously described (Dede Eren et al., 2020). Briefly, rat tenocytes and human mesenchymal stem cells cultured on tendon imprints and the Topo1018 surface were fixed at designated time points with 4% paraformaldehyde in PBS for 20 min and washed with PBS twice to remove the remaining fixative. Next, the samples were permeabilized with 0.01% Triton X-100 in PBS for 10 min. The samples were incubated with 1:100 horse serum (HS) in PBST [PBS with 0.02% Triton ×-100 and 0.5% bovine serum albumin (BSA)] for 60 min and washed with PBST. The samples were incubated overnight with monoclonal mouse IgG1 for vinculin (Sigma, V9131, 1:200) diluted in PBST and subsequently washed three times with PBST. The samples were then incubated with 1:200 goat antimouse IgG conjugated with Alexa488 (Molecular Probes, A21121) and 1:200 Phalloidin 647 (Sigma) in PBS-T for 1.5 h. Afterward, the imprints were washed three times with PBST, thereafter they were incubated for 30 min with 1:50 DAPI in PBS-T. The DAPI solution was removed, and the samples were washed three times with PBS. Then, the samples were mounted on a glass slide with Mowiol and maintained at 4°C until imaging.

Human mesenchymal stem cells cultured on binary micropatterns were washed with phosphate-buffered saline (PBS) and fixed with 3.7% paraformaldehyde (Sigma-Aldrich) for 10 min at room temperature. After washing three times, the cells were permeabilized with 0.01% Triton X-100 and blocked with 3% BSA in PBT (PBS + 0.02% Triton X-100, and 0.5% BSA) for 1 h. Afterward, the cells were incubated with the primary antibody rabbit antihuman paxillin antibody (1:200; Abcam; ab32084) dissolved in PBT overnight at 4°C. The cells were washed three times and incubated with a specific secondary goat antibody conjugated to Alexa Fluor 647 (1:400; Thermo Fisher), together with phalloidin conjugated to Alexa Fluor 568 (1:250; Thermo Fisher) in PBT for 1 h. After washing, the nucleus was counterstained with Hoechst 33258 (1:1000) for 10 min. After washing three times, the surfaces were mounted on glass cover slides with mounting media (Dako). All washing steps were performed with PBT.

### SiR-Actin Live Imaging

To visualize live actin organization, SiR-actin Kit (Spriochrome, CY-SC001) was used by following the manufacturer’s instructions. Briefly, after letting tenocytes adhere tendon imprints and flat surface for 2 hours, we replaced the culture medium with a culture medium enriched with a final concentration of 100 nM of SiR-actin probe and 1 nM of verapamil. Images were taken every 10 min with a Leica DMI8 microscope for 40 h. During imaging, the cells were inside in a humidified tissue culture chamber at 37°C with 5% CO_2_.

### Imaging and Data Analysis

Focal adhesion imaging was performed using a confocal microscope (Leica TCS SP5X). The images were taken at ×63 magnification. When images were taken at z-stacks, section thickness varied between 0.5 and 3 μM. Maximum projection was used when performing z-projection. Focal adhesions were identified and differentiated from the non-specific staining and background fluorescence based on their brightness and shape. Their length was measured by using Image J (Schneider et al., 2012) with the measure plugin. Per condition, focal adhesions of 20–30 cells were measured. The cell shape was analyzed using CellProfiler version 3.1.8 (Carpenter et al., 2006) employing custom-made pipelines. Briefly, after the correction of illumination of the two channels, the nuclei were identified as primary objects by global minimum cross-entropy thresholding from the DAPI image channel. Subsequently, cell shape was determined as the secondary object by propagating and again applying global minimum cross-entropy thresholding from the phalloidin image channel. Per condition, around 10–15 images were used, and median value of cells per image was used for data analysis. Figures were prepared by using InkSpace, and graphs were prepared by using GraphPad Prism version 8.0 (GraphPad Software, Inc., San Diego, CA, United States).

### Statistical Analysis

**FIGURE 1:**
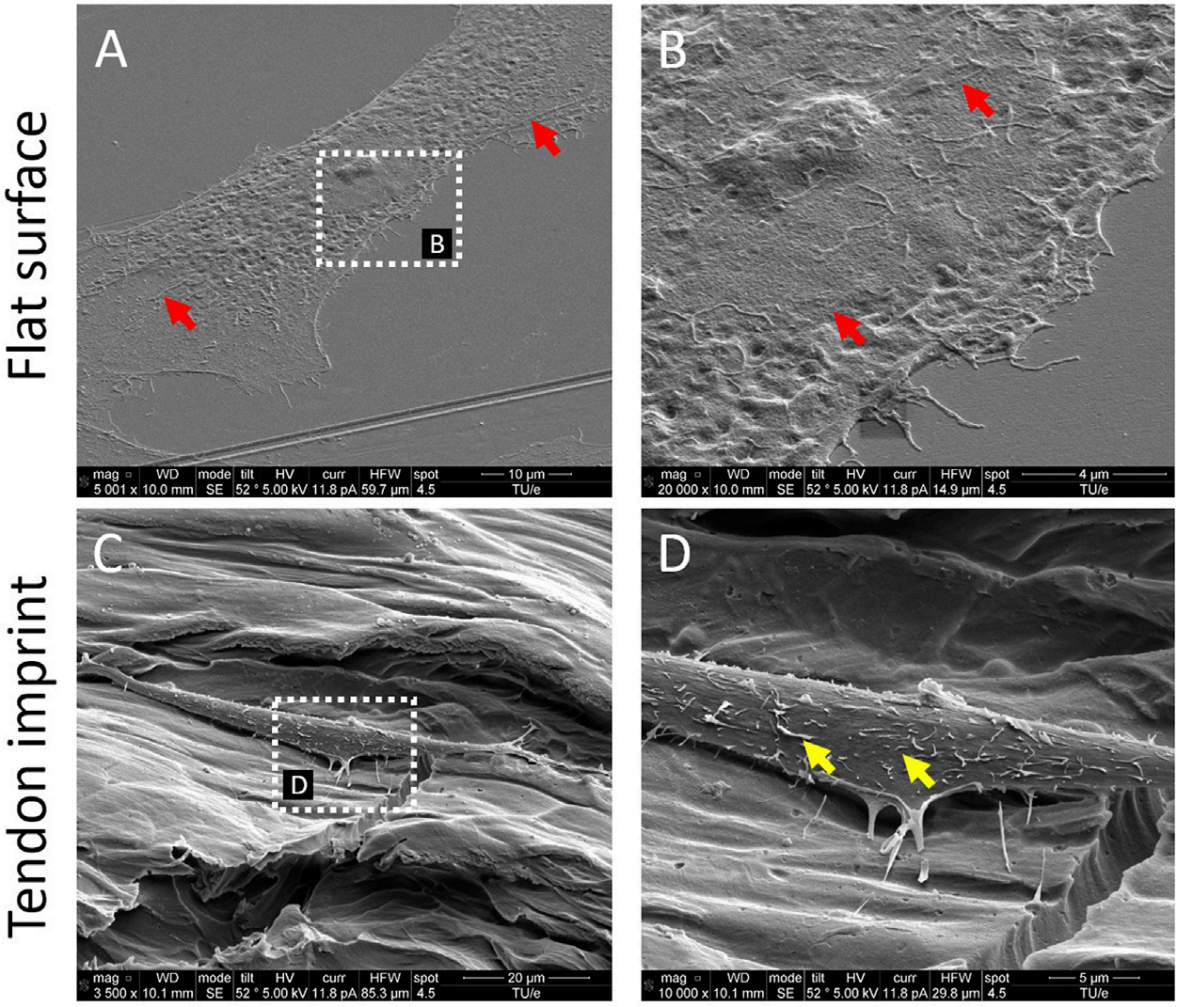
Tendon imprints induce cell shape changes. **(A)** Tenocyte on a flat surface. **(B)** Higher magnification image of tenocytes on the flat surface that illustrates cell adhesion points in higher resolution. Red arrows indicate stress fibers and yellow arrows indicate membrane invaginations sticking from the plasma membrane. **(C)** Tenocyte on the tendon imprint. **(D)** Higher magnification image of tenocytes on tendon imprint. Filopodia are observed on both substrates.

All statistical analyses were performed by using GraphPad Prism version 8.0 (GraphPad Software, Inc., San Diego, CA, United States). Prior to data analysis, each data was tested for normality by performing a Shapiro–Wilk test. A two-way analysis of variance (ANOVA) was performed to determine the statistical significance in Figures 1, 3, 4. One-way ANOVA was performed to determine the statistical significanceFigures 6, 7. Tukey’s multiple comparison test was used to determine which group differences are statistically significant. The student’s *t*-test was carried out for Figure 9. Significance set at *p* < 0.05 to determine the significance between means. All quantitative data represented in this study are based on biological triplicates (*N* = 3) unless stated otherwise.

## Results

### Tenocytes Display More Punctate Attachment on Tendon Imprint Surfaces

To investigate the effect of the topographical environment on attachment, we cultured primary rat tenocytes on polystyrene (PS) tendon imprints and flat control surfaces for 24 h (Figure 1A). Tendon imprints reproduce the natural crimped architecture of collagenous tendons, resulting in hills and valleys. Due to its heterogeneous nature, the depth of the valleys varies between 5 and 20 µM, and the frequency of the valleys and hills varies. The heterogenic nature of the tendon imprint landscape is reflected in the cell morphologies that we observe. Yet, we do see clearly that tenocytes on imprints displayed morphology similar to *in vivo* tenocytes with an increased aspect ratio (Figure 1C), that is, they are less wide than a typical flat surface cultured tenocyte. Also, tenocytes appeared to be flatter on the flat surface (Figure 1B) while cells on the tendon imprint were higher. The length of the cells appeared to be the same. On the flat surface, we observed filamentous (F)-actin stress fibers reaching from one end of the cells to the other, as pointed out in Figure 1A,B with red arrows, we did not observe such on the imprints. Interestingly, cells on the imprint were not in continuous contact with the surface. Rather, the cells were associated with the surface on an estimated dozen sites, whereas large parts of the cell body were not in direct contact with the surface. In contrast, cells on the flat surfaces seemed to be in contact over their full length. Furthermore, both cells exhibit filopodia yet lamellipodia were more predominant in tenocytes on the flat surface. Finally, we observed that cells on tendon imprint have membrane invaginations sticking from the plasma membrane, similar to the observations by Von Erlach et al. (2018) as illustrated with yellow arrows in Figure 1D. Overall, tendon imprint results in a 3D cell shape with fewer adhesion sites, suggesting changes in focal adhesion and actin cytoskeleton organization.

### Tenocyte Shape and Actin Response are Different on Cells on the Flat Surface and Tendon Imprint

**FIGURE 2:**
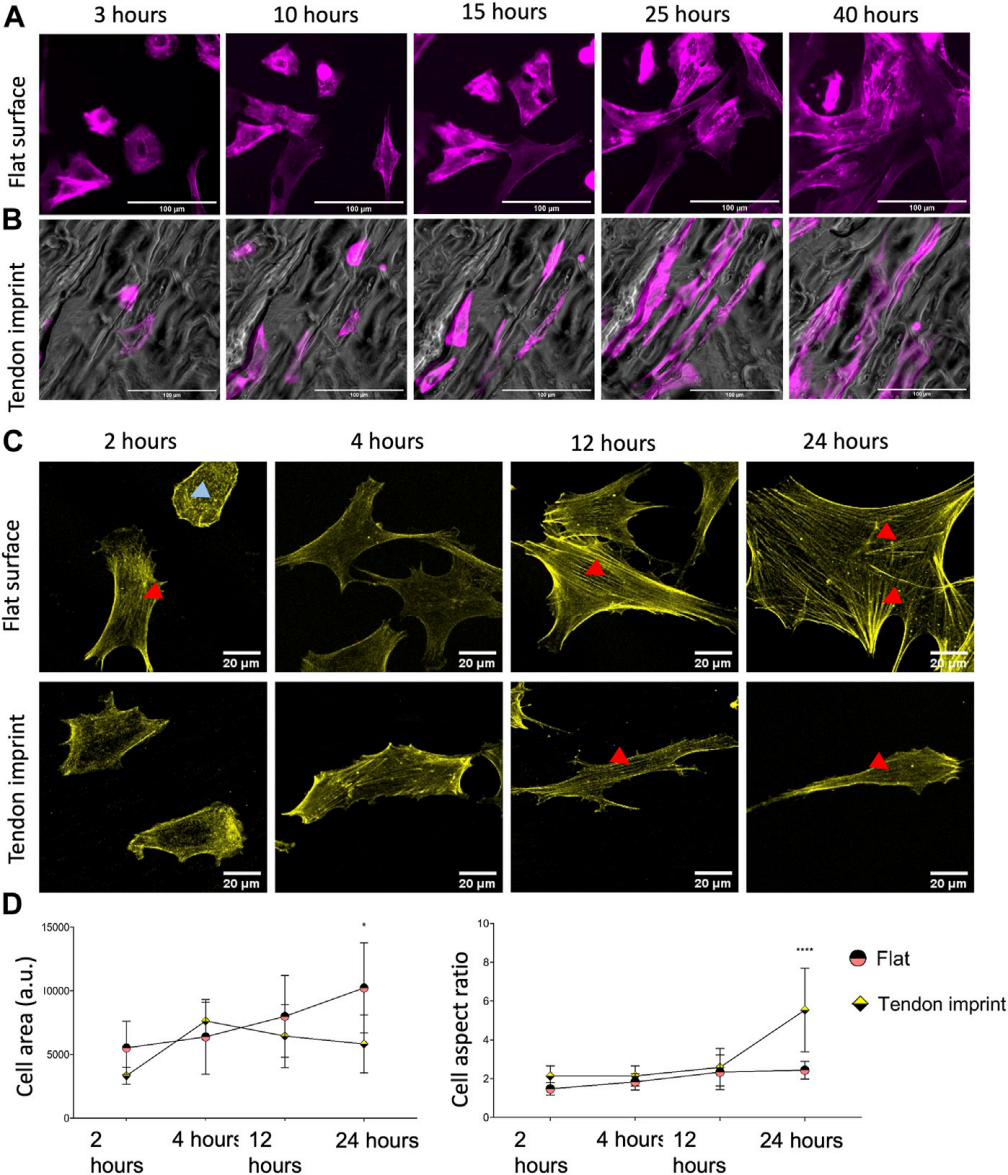
Dynamic cytoskeletal organization, cell area, and aspect ratio of tenocytes. **(A)** Snap-shots of tenocytes cultured on a flat surface, stained with SiR-actin (purple) to illustrate actin cytoskeleton and stress fibers. Scale bars represent 100 µM. **(B)** Snap-shots of tenocytes cultured on tendon imprint, stained with SiR-actin (purple) to illustrate actin cytoskeleton and stress fibers. Scale bars represent 100 µM. **(C)** Tenocytes seeded on a flat surface (top panel) and tendon imprint (bottom panel), fixed after 2, 4, 12, and 24, and stained with phalloidin (yellow). Red arrows point the ventral stress fiber and blue arrows point dorsal stress fibers. **(D)** Quantification of the cell area (left) and cell aspect ratio (right) at different time points. The area of tenocytes on the flat surface increased over time, whereas on the tendon imprint this remained at statistically similar levels after 4 h. The cell aspect ratio on the tendon imprint makes an increase after 24-h yet remained in similar values between 2 and 24-h on a flat surface. Scale bars represent 20 µM. (Error bars represent 95% confidence intervals, *p < 0.05, ****p < 0.001) (*’s correspond to comparisons between different culture substrates in the same time point). For all experiments, *N* = 3.

To verify that tendon imprints affect actin organization, we monitored the dynamics of actin organization and adaptation of cytoskeleton and cell shape on the flat surface and the tendon imprint. We stained tenocytes with SiR-actin and imaged them for 40 h (Supplementary Video S1, S2, Figures 2A,B). In cells on the flat surface at 2 h (Figure 2C), we observed dorsal stress fibers (blue arrow), non-contracting actin fibers at the periphery of the cell (Tojkander et al., 2012), and ventral stress fibers (red arrows), which are contracting actomyosin fibers located at the posterior parts of the cells and have direct attachments to focal adhesions (Tojkander et al., 2012). In recently divided cells, stress fibers are organized as cortical and transverse arcs, which are curved actin bundles that transmit contractile forces and through dorsal stress fibers (Tojkander et al., 2012). As the cell size increased, stress fibers became thicker and appeared more ventral and peripheral (red arrows, top panel) (Figure 2C). The cells displayed a variety of shapes, migrated freely in all directions, and constantly formed lamellipodia. Tenocytes on tendon imprint cells migrated preferentially in the direction of the valleys (Figure 2B, Supplementary Video S2). Furthermore, cells acquired the shape of the topography underneath approximately within 40 min after cell division. Stress fibers were observed to be only in the long axis of the cells (red arrows, bottom panel) (Figure 2C, Supplementary Video S2).

To quantify the cell area and elongation, tenocytes were fixed after 2, 4, 12, and 24 h and stained for F-actin (Figures 2C,D). On the flat surface, we confirmed stress fiber formation from 2 h on, becoming thicker as the cells became larger (Figure 2C, top panel). On tendon imprints, elongated cell morphology peaked after 24 h (Figure 2C, bottom panel), and the cell area remained significantly less compared to the flat surface (Figure 2D). Nonetheless, we observed more stress fiber formation in the cells on a flat surface than on the tendon imprint. Therefore, we conclude that there is a dynamic change in the cell area and elongation in tenocytes upon culturing them on the tendon imprint and the flat surface. Changes in cell shape seem to occur once a small number of stress fibers forms in the direction of contact guidance.

### Surface Topography Guides Stress Fiber Formation and Cell Shape in Human Mesenchymal Stem Cells

**FIGURE 3:**
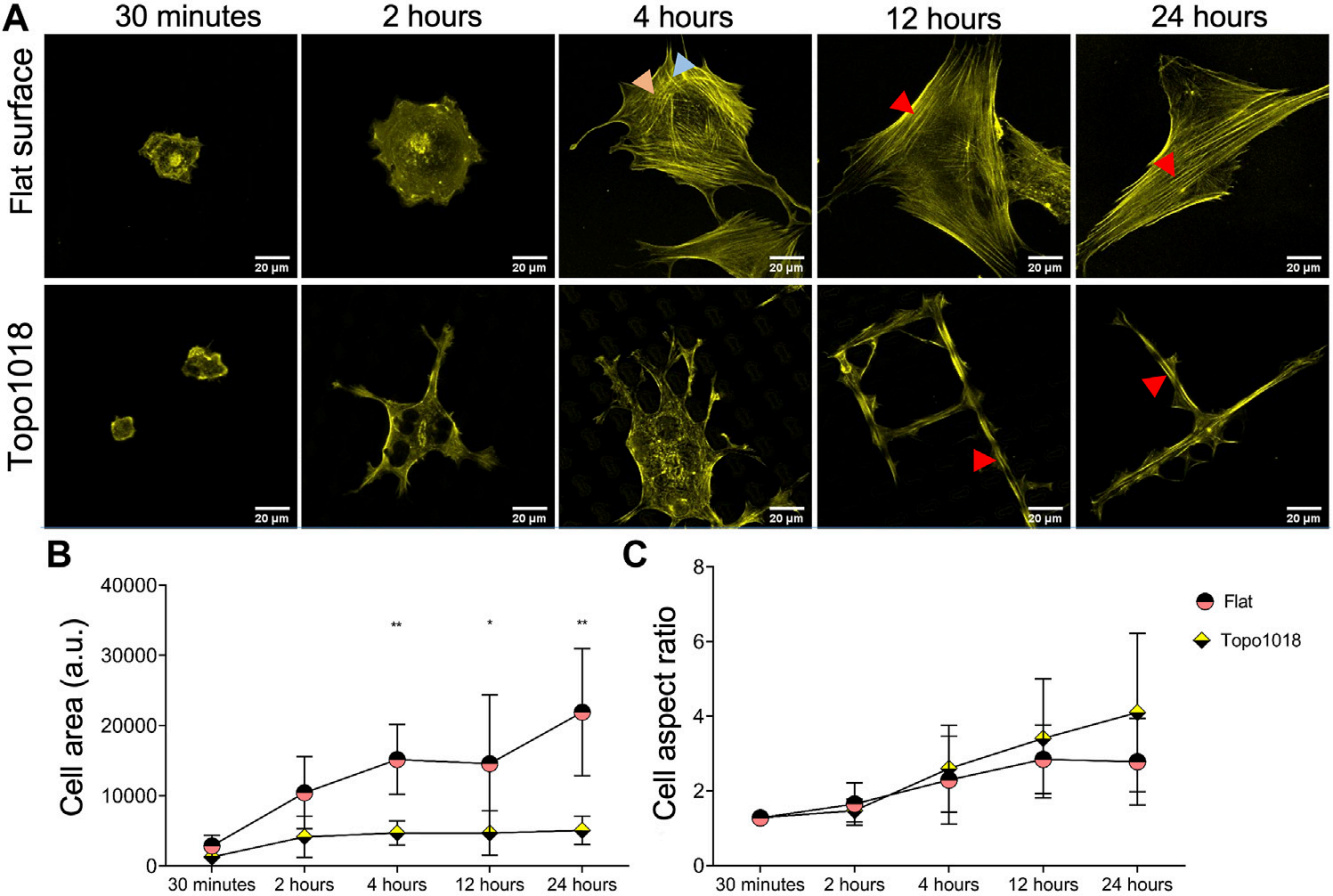
hMSC shape and cytoskeletal organization on topography 1018 and flat surfaces. **(A)** hMSCs cultured on a flat surface and a Topo1018 surface and stained with phalloidin to visualize F-actin (yellow) at 30 min, 2, 4, 12, and 24 time points. On a flat surface (top panel), transverse arcs and dorsal stress fibers are observed at 2 h. This replaces itself with thick ventral stress fibers after 4 h. On the Topo1018 surface (bottom panel), hMSCs are on top of the pillars and stress fibers are observed on the lamellipodia on the bottom of the topography at 4 h. After 12 h, cells settled between the pillars and sat between the topographies and displayed long and think stress fibers along their long axis. Scale bars represent 20 µM. Red arrows point the ventral stress fiber, blue arrows point dorsal stress fibers, and orange arrows point the transverse arcs. **(B)** Quantification of the cell area over time showed that after 4 h, the difference between the cell area between hMSCs on a flat surface and a Topo1018 surface becomes significant. **(C)** Quantification of cell aspect ratio suggests that on the Topo1018 surface there is a steady increase in cell elongation while on a flat surface it stabilized after 12 h (Error bars represent 95% confidence intervals, *p < 0.05, **p < 0.01, and ****p < 0.001) (*’s correspond to comparisons between different culture substrates in the same time point). For all experiments, *N* = 3.

We previously noticed that human bone-derived mesenchymal stem cells (hMSCs) dynamically adapt the shape and physiology upon interaction with Topo1018, which is a platform composed of uniquely designed microtopographies and was previously demonstrated to create elongated cell morphology (Vermeulen et al., 2020) similar to the tenocytes on the tendon imprint. To see if changes in actin organization run in parallel to those in cell shape, as it did in tenocytes, we seeded hMSCs on a flat surface and Topo1018 and stained the actin cytoskeleton after 30 min, 2-h, 4-h, 12-h, and 24-h (Figure 3A). The cell area was similar 30 min after seeding but was significantly different between the flat surface and Topo1018 surface already after 4 h. On the flat surface, the cell area kept increasing as the cells were spreading, whereas the Topo1018 cell area stabilized after 2 h (Figure 3B). The aspect ratio remained similar from 4 h onward in hMSCs on the flat surface, but on the Topo1018 surface, there was a steady increase (Figure 3C), that is, the cells became thin and elongated. Interestingly, cells on Topo1018 were mainly observed on top of the pillars at 30 min, 2 h, and 4 h, but after 12 and 24 h, they were mostly confined between the pillars. On the flat surface, hMSCs possess transverse arcs (orange arrows) and dorsal stress fibers (blue arrows), as we observed previously in tenocytes. From 4 h onward, we observed that the thickness of stress fibers increased, and the actin cytoskeleton was mainly formed by ventral stress fibers (red arrows), reaching from one end of the cell to the other (Figure 3A-top panel). On Topo1018, the switch in the location from pillar top to the valleys coincided with the appearance of stress fibers in the main axis of the cells (see 4 h vs. 12 h in Figure 3A). After 24 h, the number of stress fiber was smaller in hMSCs on Topo1018 surfaces than on flat surfaces.

### Maturation of Focal Adhesion Differs Between Cells on Flat Surfaces and Topographies

**FIGURE 4:**
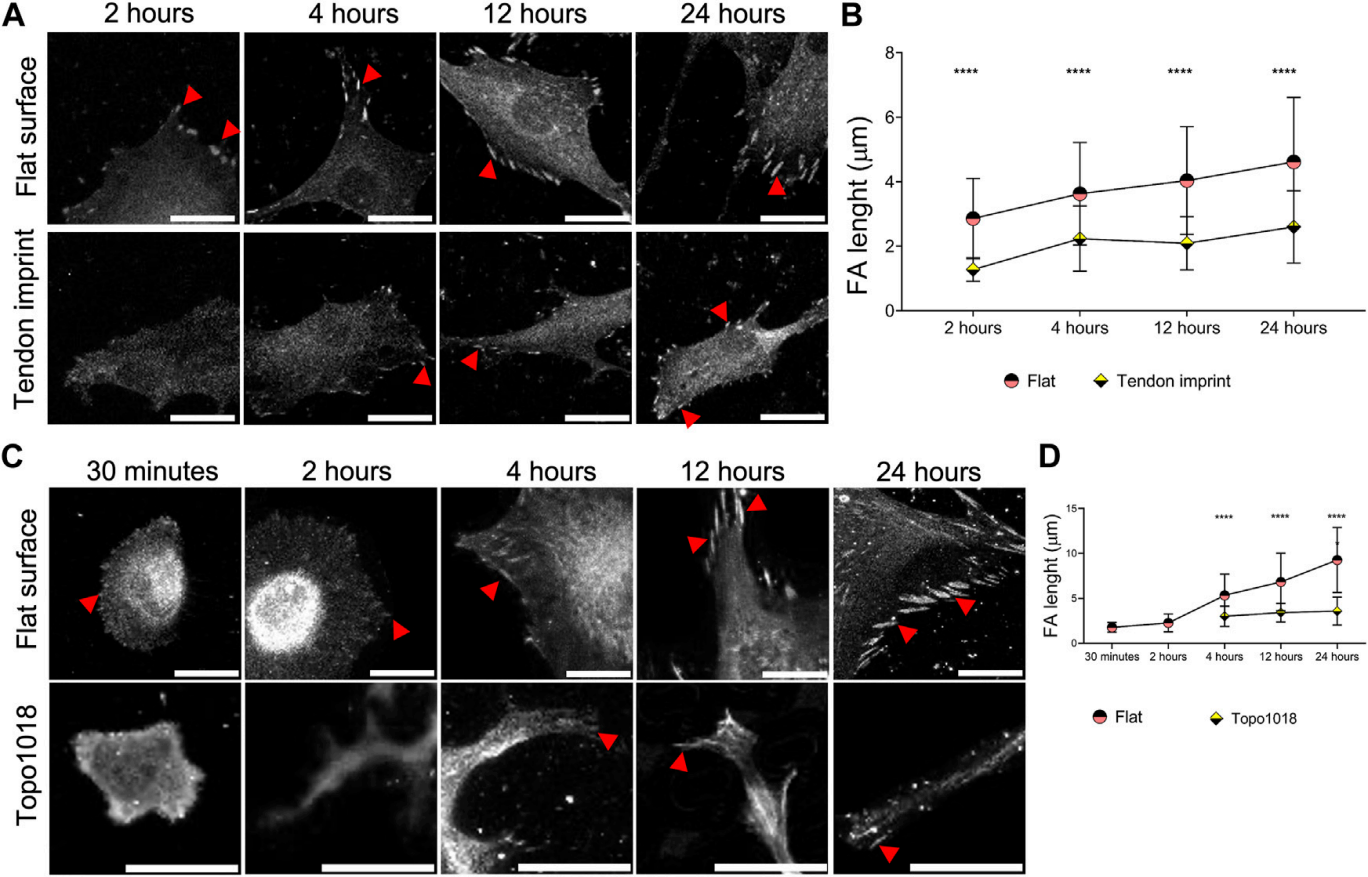
Surface topography modulates the maturity and position of focal adhesions. **(A)** Rat tenocytes cultured on a flat surface and the tendon imprint for 2, 4, 12, and 24 h, and stained for vinculin (fair gray color) and pointed with red arrows. Scale bars represent 20 µM. **(B)** Quantification of focal adhesion length indicates that on a flat surface, as the cells become larger, focal adhesions become longer indicating their maturation. Tenocytes on the tendon imprint, focal adhesion length is significantly smaller compared to the flat surface at the all-time point. **(C)** hMSCs cultured on a flat surface and a Topo1018 surface for 30 min, 2, 4, 12, and 24 h, and stained with vinculin (fair gray color) and pointed with red arrows. Scale bars represent 20 µM. **(D)** Focal adhesion length increases steadily in cells cultured on a flat surface. On the Topo1018 surface, we did not observe focal adhesions after 30 min and 2 h, and from 4 h onward, their length remained at similar levels and significantly shorter compared to their flat surface counterparts. (Error bars represent 95% confidence intervals, ****p < 0.001) (*’s correspond to comparisons between different culture substrates in the same time point). For all experiments, *N* = 3. (FA = focal adhesion).

The effect of topography on actin organization suggests that the contact of cells with the surface is different. To investigate this, we seeded rat tenocytes on the tendon imprint and the flat surface and stained them for vinculin to measure focal adhesions length (Figures 4A,B). We observed a steady increase in focal adhesions length in tenocytes on flat surfaces, consistent with the maturity of the stress fibers, and an increase in the cell area (Figures 4A,B). On tendon imprints, we see only very few focal adhesions at early time points and more after 24 h. However, focal adhesions length does not seem to increase, which is in line with stress fiber formation and the cell area. We observed a similar, even more, pronounced response on Topo1018 (Figures 4C,D) 30 min and 2 h after seeding, we did not observe focal adhesions and those that appeared later are at the end of stress fibers at the bottom, confined between the pillars, but not on the pillars (Figure 4C). The focal adhesion length is consistently lower in hMSCs on Topo1018 than on the flat surface (Figure 4D). These data show that the maturation of stress fibers and focal adhesions is in sync with the topographical information provided by the surface on which they grow.

### Spatial Organization of Adhesive Islands Steers Focal Adhesion Formation, Actin Cytoskeleton, and Cell Shape

**FIGURE 5:**
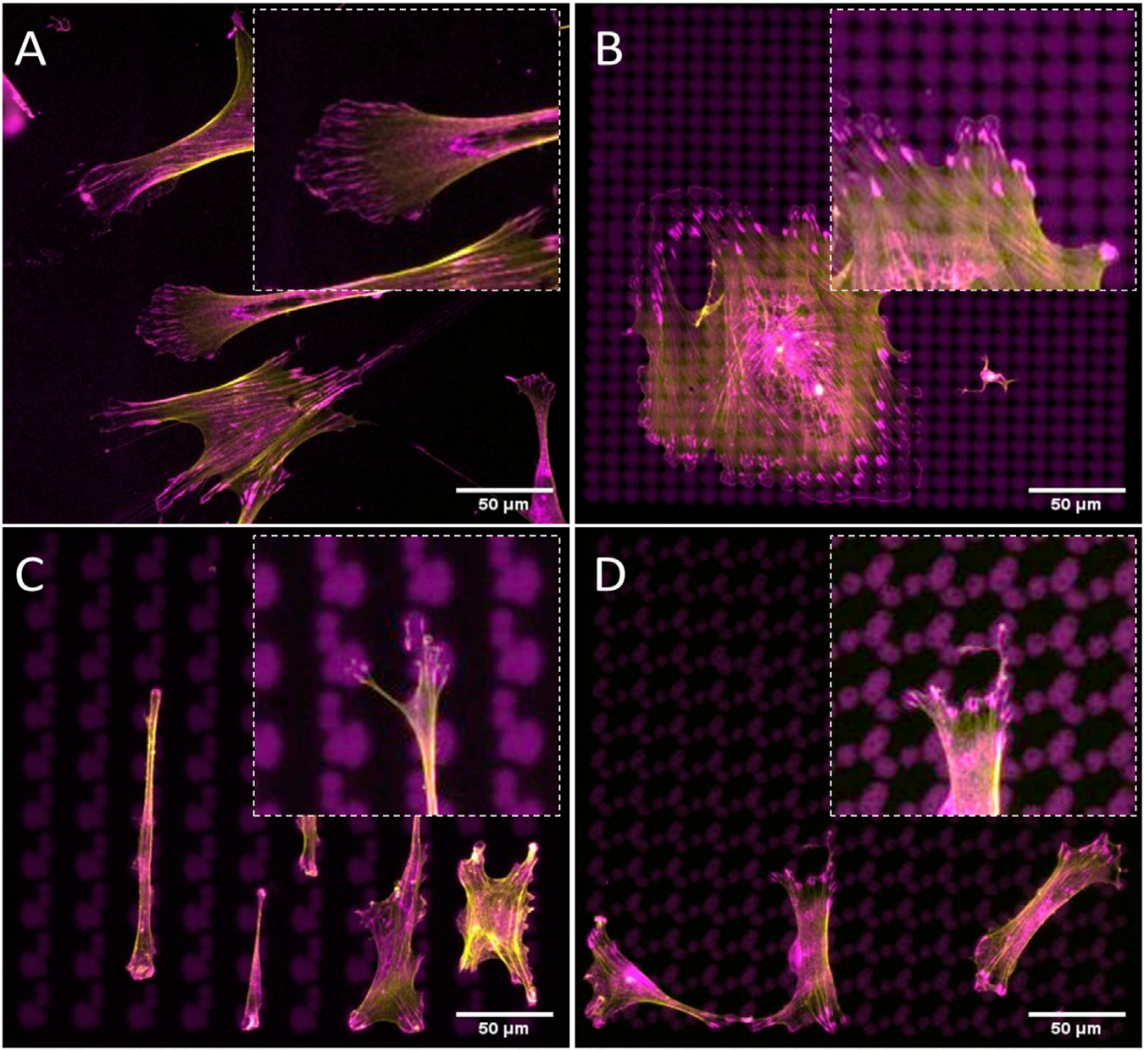
Cell shape can be controlled by the spatial organization of adhesive islands. hMSCs are cultured surfaces fully covered with **(A)** continuous lawn of RGD peptides or **(B–D)** 2D micropatterns for 4 h and stained with phalloidin to visualize F-actin (yellow) and paxillin (magenta). Adhesive islands are presented in magenta. Scale bars represent 50 µM.

We next wondered whether contact guidance is necessary for guiding cell shape, that is, if the confinement imposed by the third dimension of the topography is required for the spatial organization of focal adhesions and F-actin that we observe on topographies. To this end, we used the Galapagos library of binary adhesive micropatterns, in which 2,074 different islands of RGD peptides are produced on a glass substrate based on the pillar design of the TopoChip (Tuvshindorj et al., 2022). hMSCs were cultured for 4 h on the Galapagos chip and stained for F-actin and paxillin (Figure 5). On the control surface, covered with a continuous lawn of RGD peptides, lamellipodia were observed randomly and ventral stress fibers are associated with long focal adhesions, indicating high cell tension (Figure 5A). When the cells are seeded on small islands which are close to each other (100 full islands in 65 × 65 μM area, zoom in box at the right top corner), we observed that the cells tend to generate more adhesive sites and display a spread morphology and high numbers of lamellipodia (Figure 5B). We observed thick ventral stress fibers with long focal adhesions at each end, indicating high tension in the cells. On small islands separated by 10–15 µM of non-adhesive surface (nine full islands (Figure 5C) and 16 full islands (Figure 5D) in 65 × 65 μM area), the cells anchored themselves on a limited number of islands, with focal adhesions having firm ground on the islands and stress fibers attached to them. We also searched the Galapagos chip for cells with a high aspect ratio resembling tenocytes. We found them on surfaces, in which islands were relatively close together in one direction but separated in the other direction (Figures 5C,D). On these islands, not only the shape was like tenocytes on the tendon imprint but we also observed that focal adhesions were located at the extremities of the cells, with a smaller number of stress fibers than on the fully RGD covered surface. This demonstrates that cell shape is also guided by the availability of adhesive sites, *via* contact guidance, and the possibility to build stress fibers.

### Interfering With Integrin-Mediated Cell Adhesion Alters Cell Shape and Focal Adhesion Length

**FIGURE 6:**
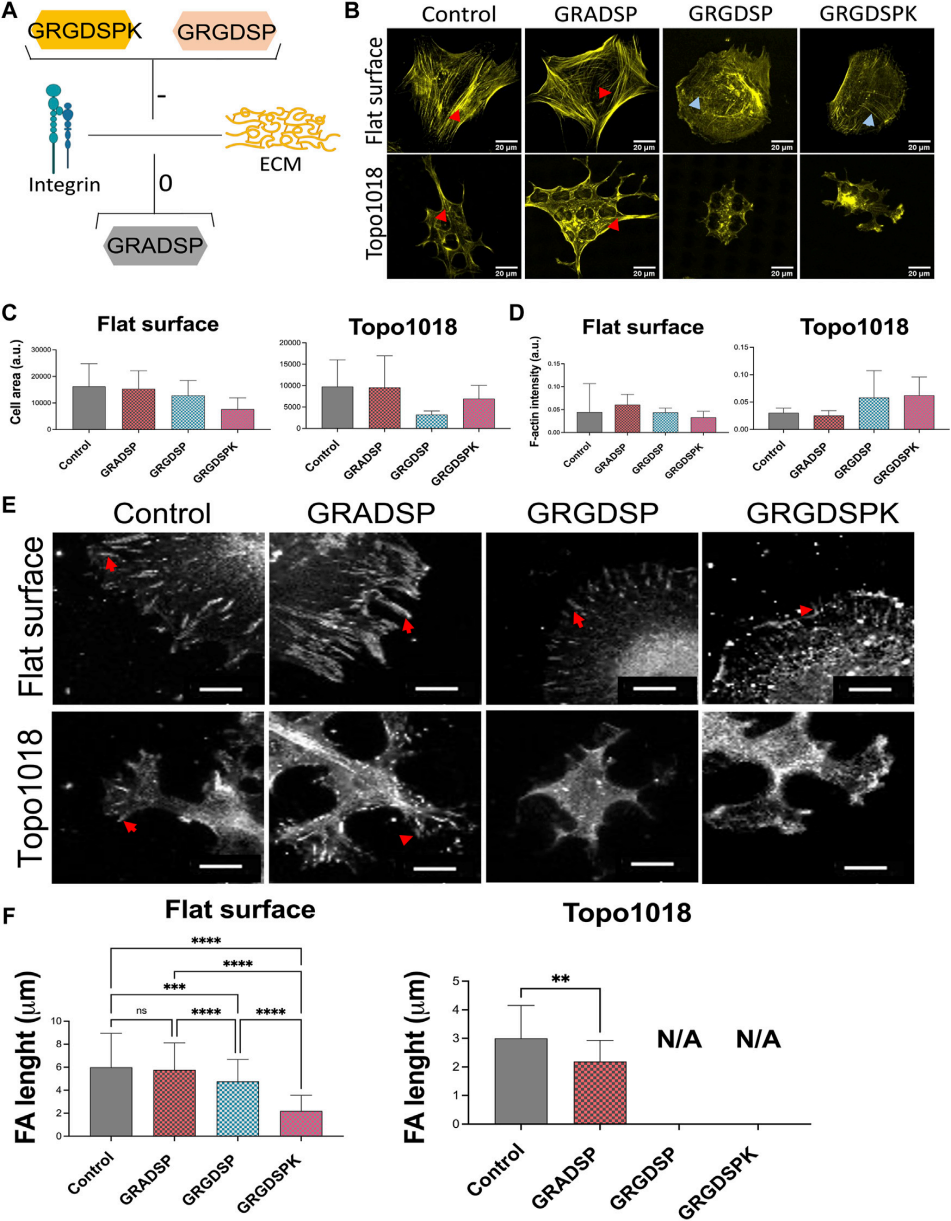
Interfering with integrin-mediated cell adhesion *via* RGD peptides leads to changes in stress fibers and focal adhesion length. **(A)** Illustration of the action mechanism of the peptides used in this study: Gly-Arg-Gly-Asp-Ser-Pro-Lys (GRGDSPK) and Gly-Arg-Gly-Asp-Ser-Pro (GRGDSP) to inhibit integrin-mediated cell adhesion and used Gly-Arg-Ala-Asp-Ser-Pro (GRADSP) as a negative control. [**(B)**-Top panel] On a flat surface, control and GRADSP treatment resulted in similar cell shape and stress fiber appearance. GRGDSPK and GRGDSP treatments altered the stress fiber pattern to a more dorsal stress fiber appearance. Scale bars represent 20 µM. [**(B)**-Bottom panel] On the Topo1018 surface, we observed changes in cell shape and stress fiber structure. Scale bars represent 20 µM. Blue arrows point the dorsal stress fibers and red arrows point the ventral stress fibers. **(C)** Quantification of the cell area of cells cultured on the flat and Topo1018 surfaces and treated with peptides. **(D)** Quantification of F-actin intensity of cells cultured on flat and Topo1018 surfaces and treated with peptides. [**(E)**-Top panel] Focal adhesions of hMSCs cultured on a flat surface (gray) are pointed with a red arrow. In control groups, the length of focal adhesions remains similar, yet GRGDSPK and GRGDSP treatment resulted in smaller focal adhesions. Scale bars represethe nt 10 µM. [**(E)**-Bottom panel] Focal adhesions of hMSCs cultured on the Topo1018 surface (gray) are pointed with a red arrow. In control groups, the length of focal adhesions remains similar; however, we did not observe focal adhesions on the GRGDSPK- and GRGDSP-treated groups. Scale bars represent 10 µM. Vinculin antibody staining was performed to visualize the FAs. **(F)** Quantification of focal adhesion length of cells cultured on the flat and Topo1018 surfaces and treated with peptides. (Error bars represent 95% confidence intervals, **p < 0.01). For all experiments, *N* = 3. (FA = focal adhesion).

The results so far show that cell shape depends on the spatial organization of the surface on which the cells grow. Next, we wanted to investigate how cell shape depends on the organization of the cell’s adhesive machinery, by blocking the interaction between integrins and the extracellular matrix. We grew hMSCs on surface Topo1018 and a flat control for 4 h, the earliest time point at which we detect focal adhesions on the Topo1018 surface and the cell size are significantly different. The culture medium was supplemented with peptide Gly-Arg-Gly-Asp-Ser-Pro-Lys (GRGDSPK) or Gly-Arg-Gly-Asp-Ser-Pro (GRGDSP), which bind to integrins and thus block integrin-ECM interaction. Peptide Gly-Arg-Ala-Asp-Ser-Pro (GRADSP) was used as a negative control (Figure 6A). On the flat surface, stress fibers appeared of similar thickness and pattern in control (no RGD peptide supplemented) and GRADSP condition, the cell area, and F-actin intensity were the same (Figures 6C,D). Similarly, on the Topo1018 surface, the cell area and F-actin intensity remain similar between the control and GRADSP groups (Figures 6C,D). In the presence of the integrin-binding peptides GRGDSPK and GRGDSP however, hMSCs on the flat had more dorsal stress fibers (blue arrows), lost their lamellipodia, and became more rounded. Peptide GRGDSP also resulted in cells with a smaller area (Figure 6B, top panel), and cells treated with GRGDSPK had smaller focal adhesions, demonstrating the biological activity of the peptides. Remarkably, treatment with either peptide leads to complete loss of focal adhesions in 1018. Increased F-actin intensity was observed (Figures 6B,D) although the ventral stress fibers reaching from one end to the other end (red arrows), appeared to be lost upon RGD peptide supplementation (Figure 4B, bottom panel). However, this did not affect the cell size. Overall, these results indicate integrin-blocking RGD peptides have a drastic effect on focal adhesions organization on topographical surfaces. Furthermore, interfering with cell adhesion indeed changed the organization of F-actin.

**FIGURE 7:**
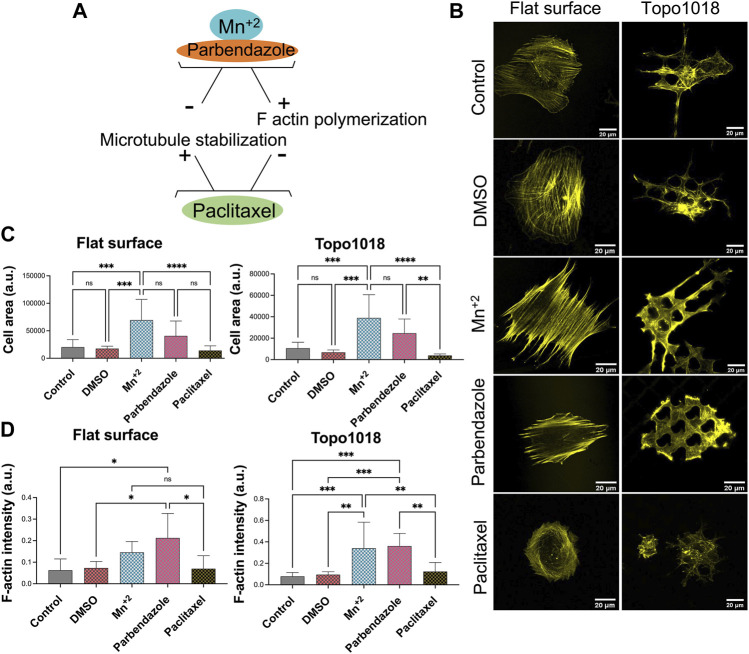
Small molecules that influence integrin signaling, actin polymerization, and microtubule stability affected the actin cytoskeleton. **(A)** Illustration of the action mechanism of the small molecules used in this study: Mn^2+^ to activate integrin signaling and induce F-actin polymerization, parbendazole to induce F-actin polymerization and degradation of microtubules, paclitaxel to compromise F-actin polymerization and stabilize microtubule assembly [**(B)**- Left panel] Treatment with Mn^2+^ and parbendazole increased the thickness of the ventral stress fibers while paclitaxel treatment lead to the formation of thinner stress fibers compared to control groups. Scale bars represent 20 µM. [**(B)**-Right panel] On the Topo1018 surface, similar to the flat surface, we observed that stress fibers become thicker, and cells attached to the bottom of the surface upon Mn^2+^ and parbendazole treatment. Scale bars represent 20 µM. **(C)** Quantification of the cell area of cells cultured on the flat and Topo1018 surfaces and treated with small molecules. **(D)** Quantification of F-actin intensity of cells cultured on the flat and Topo1018 surfaces and treated with small molecules. (Error bars represent 95% confidence intervals, *p < 0.05, ***p < 0.005). For all experiments, *N* = 3.

**FIGURE 8:**
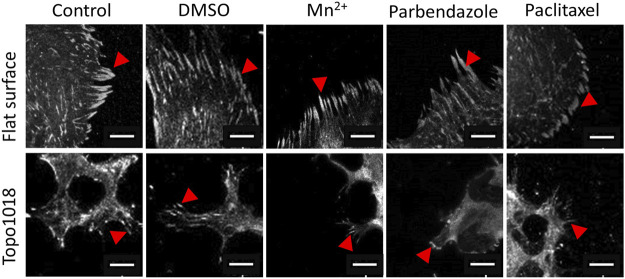
Small molecule-targeting microtubule stabilization resulted in shorter focal adhesions. We cultured hMSCs on the flat and Topo1018 surfaces and treated them with small molecules targeting integrins (Mn^2+^), F-actin, and microtubules (paclitaxel and parbendazole), and we stained them with vinculin to visualize focal adhesions and pointed them with red arrows. Scale bars represent 10 µM. For all experiments, *N* = 3.

Next, we did the opposite experiment, that is, increase integrin affinity to ECM, by adding Mn^2+^ to the culture medium in which hMSCs grew on flat and 1018 surfaces (Figure 7). On flat, we observed a strong increase in the cell area and a profound increase in F-actin intensity. Mn^2+^ treated cells have very thick stress fibers, indicating that they induce integrin/ECM interaction and thus affect focal adhesion organization and cell shape. (Figure 7B, left panel, Figures 7C,D). On 1018 surfaces too, Mn^2+^ treatment led to the formation of larger cells with thicker stress fibers (Figure 7B, right panel, Figures 7C,D), demonstrating a correlation between the cell area and strength of adhesion of the cells to the surface. However, despite the visible change in the cell area and F-actin organization, the focal adhesion size did not noticeably change upon the addition of Mn^2+^ on either flat or the 1018 surface (Figure 8).

### Actin Organization is Essential for Cell Shape

We next questioned how increasing or reducing actin polymerization affects focal adhesion formation, stress fiber formation, and cell shape (Figure 7). As expected, treatment of hMSCs grown on flat surfaces with the microtubule inhibitor parbendazole led to increased actin polymerization with very pronounced stress fibers (Figure 7B), as reported previously (Brum et al., 2015). The compound paclitaxel compromises F-actin polymerization and has the opposite effect: cells have fewer stress fibers and the cell area is reduced 0.7-fold compared to untreated control and 0.35-fold compared to parbendazole treated cells on the flat surface (Figure 7C). On topography 1018, parbendazole treatment resulted in the intensity in the filamentous actin intensity (Figure 7D). Similar to our observations on the flat surface, paclitaxel treatment of the cells on Topo1018 surface resulted in a 0.14-fold decrease in the cell area compared to parbendazole-treated counterparts and a 0.37-fold decrease compared to untreated controls (Figures 7C,D). Furthermore, paclitaxel treatment in both flat and Topo1018 surfaces resulted in the change of the organization and phenotype of stress fibers. Contrary to parbendazole treatment, which leads to the formation of thick ventral stress fibers, paclitaxel treatment results in the formation of mainly thin dorsal stress fibers and transverse arcs. Finally, we did not notice a change in focal adhesion length upon parbendazole or paclitaxel treatment (Figure 8). These results suggest that manipulation of actin organization can be independent of the maturation of the focal adhesions.

### Cells That are Obtained From Tissues With Different Stiffnesses Display Similar Responses on Tendon Imprints

**FIGURE 9:**
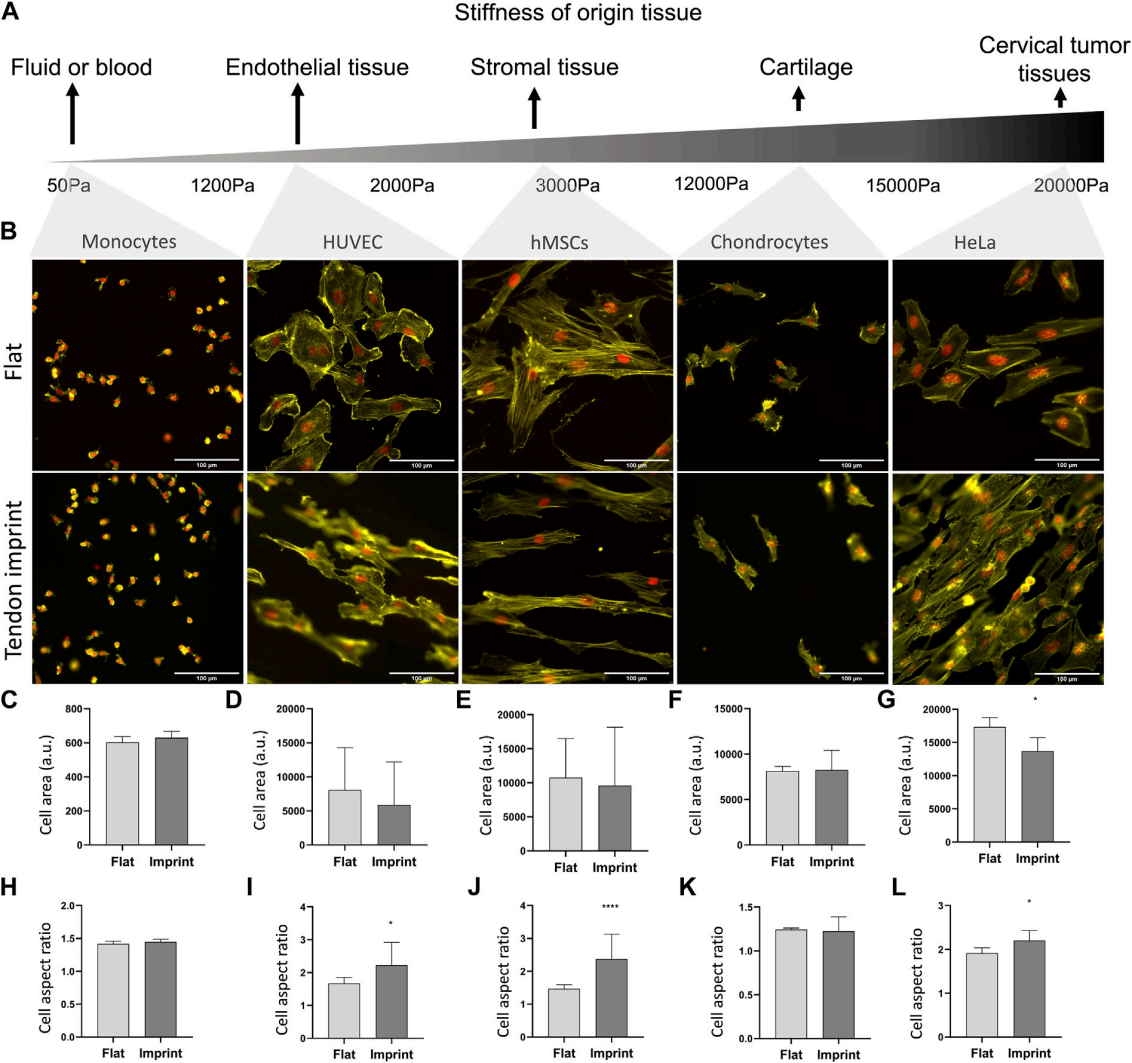
Different cells on tendon imprint undergo similar changes in the cell area and aspect ratio. **(A)** Panel illustrating the stiffness of tissues from which the monocytes, HUVEC, hMSCs, chondrocytes, and HeLa cells originate. From left to right, tissue stiffness increases. **(B)** Phalloidin (yellow) and DAPI (red) staining of monocytes, HUVEC, HMSCs, chondrocytes, and HeLa cells cultured after 24 h of culture on a flat surface and the tendon imprint. Scale bars represent 100 µM. **(C–G)** Quantification of the cell area remained in similar values in all cells except for HeLa cells on the tendon imprint. **(H–L)** Quantification of cell aspect ratio shows that the tendon imprint leads to a significant increase in the cell aspect ratio of the hMSCs, HeLa, and HUVEC cells (Error bars represent 95% confidence intervals, *p < 0.05, ****p < 0.001). For all experiments, *N* = 1.

So far, we manipulated cell adhesive properties by changing the surface or by biochemically interfering with the adhesion and actin cytoskeleton. An alternative approach to investigate the effect of topography on cell shape is to culture cells isolated from tissues with reported different stiffnesses and adhesive properties (Engler et al., 2006; Cox and Erler, 2011; Dede Eren et al., 2021b) and observe their response. For this, we seeded selected a range of cell types whose origin tissues have different stiffness, namely monocytes from blood, human umbilical vein endothelial cells (HUVEC), hMSCs from human bone marrow, chondrocytes from cartilage, and HeLa cells from cervical cancer tissue (Figure 9A). All cells adhered well to the surfaces, with the clear formation of stress fibers, except for monocytes that did adhere to the surface but did not spread. Monocytes are the smallest cell type that we selected with the least adhesive properties and upon culturing on the tendon imprint, cell shape did not change as the cell size is too small to detect the topographical cues on the tendon imprint (Figures 9B,C,H). HUVEC and chondrocytes have similar cell area (Figures 9D,F) and the contact guidance was observed. However, HUVEC displays an increase in the cell aspect ratio, and the cell area remain unchanged in both conditions (Figures 9B,D,I). hMSCs and HeLa cells are the largest cells and again orient themselves based on the topographical cues underneath (Figure 9B). Furthermore, HeLa cells, cell area, and aspect ratio changed, indicating that the cell area is an important parameter to organize their actin cytoskeleton.

## Discussion

Previously, we demonstrated that mesenchymal stem cells (Vicente-Manzanares et al., 2009; Sit and Manser, 2011; Xu et al., 2011; Maharam et al., 2015; Hulshof et al., 2017; Madhurakkat Perikamana et al., 2018; Vermeulen et al., 2020; Vermeulen et al., 2021), tenocytes (A. Dede Eren, E.D. Eren et al., 2021; A. Dede Eren, Vasilevich et al., 2021; Vermeulen et al., 2019), and pre-hypertrophic chondrocytes (Le et al., 2017) actively organize their cytoskeleton upon exposure to topography and described its phenotypic consequence on differentiation, phenotype maintenance, proliferation, and metabolism. In this manuscript, we zoom into the cell-biomaterial interface. We describe how rat tenocytes and human mesenchymal stem cells adopt their focal adhesions on surfaces with different material properties, and how this reflects on the cytoskeletal organization of the cells. We demonstrate that the dynamic organization of the actin cytoskeleton starts very early upon cell attachment, and the difference in cell shape becomes more pronounced over time. We provide further evidence on the link between focal adhesion maturation and changes in the actin stress fiber profile and emphasize this link by interfering with cell adhesive properties. Overall, the results reported in this manuscript support our hypothesis regarding the involvement of adhesion molecules in cell morphology and further expand the knowledge on the dynamic adaptation and organization of the cell-biomaterial interface.

Dynamic organization of the actin cytoskeleton to adapt to the surrounding extracellular matrix occurs immediately after cell attachment. Curtis, (1964) used interference reflection microscopy to visualize changes in the shape of embryonic chick heart fibroblasts early upon cell attachment. Pierres et al. (2008) illustrate that initial cell attachment occurs *via* the formation of small protrusions in a “tiptoe-like” manner to probe the surrounding space, and full attachment occurs after tens of seconds. Similarly, fibroblasts organize their shape on fibronectin patterns *via* contact guidance within 30 min (Ramirez-San Juan et al., 2017) but significant topography-guided differences in cell morphology take up to hours in endothelial cells (Sales et al., 2017) and human osteosarcoma-derived cells (Davidson et al., 2009). These results are in line with our findings that rat tenocytes adapt to the tendon topography early upon attachment yet differences in cell elongation and area become only apparent after 24 h, which coincide with the moment that prominent actin stress fibers become visible. We reported similar results when rat tenocytes (Vermeulen et al., 2019) as well as hMSCs was cultured on another surface topography selected from TopoChip screen, after 24 h of culturing, cells become more elongated (Vermeulen et al., 2020). Interestingly, it took hMSCs only 4 h to acquire a significant difference in the cell area on microtopographies. This suggests that adaptation of cell shape is highly dependent on the cell type and topographical properties. Monocytes, for instance, may be too small to perceive contact guidance provided by the tendon imprint in this manuscript, whereas we saw clear changes in cell shape previously on some TopoChip-derived topographies (Vassey et al., 2020). Tong et al. (2012) reported that fibroblast-like cells adapt their cytoskeleton and become more elongated in response to a tendon biomimetic surface topography compared to epithelial-like cells. In their article, in contrast to our results, Hela cells did not show contact guidance. It is unclear what causes this difference.

As the cues from the surrounding microenvironment to the actin cytoskeleton are mediated through focal adhesions, we hypothesized that the dynamic alterations in cell shape and stress fiber phenotype is associated with focal adhesion maturation. It is known that larger focal adhesions indicate higher cellular tension and come with thick ventral stress fibers (Romero et al., 2020). For instance, epithelial-like cells spread more on smooth surfaces and created longer focal adhesions compared to those on rough surfaces (Baharloo et al., 2005), indicating stronger adhesion and cellular tension on smooth surfaces. Abagnale et al. demonstrated that pluripotent stem cells (Abagnale et al., 2017) and hMSCs (Abagnale et al., 2015) on aligned topographies have a higher aspect ratio, smaller cell size, and smaller focal adhesions while cells on flat surfaces have the larger surface area and focal adhesions. An interesting study by Yang et al. (2020) showed that focal adhesions are not necessarily biggest on flat surfaces. They showed that in hMSCs, the size of focal adhesions per cell become 275 and 260 μm^2^ when the distance between the microgrooves are 0.5 and 3 μM, respectively. The area of focal adhesions was measured around 150 μm^2^ on a flat surface, and microgrooves with distances of 10 μM and 30 μM and focal adhesion size correlated with higher cell stiffness (Yang et al., 2020). We observe this link between the cell area and focal adhesion maturation in both rat tenocytes and hMSCs. Over time, parallel to the increase in the cell size, focal adhesion length increases. Furthermore, we provide further evidence on the link between stress fiber and focal adhesion maturation. As the cells grow and spread on the flat surface, the tension in the cells increases, and this is balanced with the maturation of the focal adhesions, therefore, supporting the cellular tensegrity model (Wang et al., 2001). On topographies, considering that cell spreading is already limited by the surrounding topography, apparent ventral stress fibers, and the length of focal adhesions decrease, cells have less tension in their cytoplasm.

The strength of the adhesion between ECM and integrins determines cell shape and stress fiber formation, and here, we provide further evidence by treating hMSCs with RGD peptides. Essentially, RGD is a peptide sequence found in cell adhesive proteins (e.g. fibronectin and vitronectin) and binds to specific integrins, such as integrin β1, β3, and β5 subunits, and ultimately influence the actin cytoskeleton *via* focal adhesion complexes (Lamers et al., 2012). The influence of RGD peptides on the strength of cell adhesion can be measured by using atomic force microscopy (AFM). For instance, AFM was employed to measure the adhesion strength of zebrafish primary mesendodermal progenitors to fibronectin substrates and it was reported that when cells were treated with soluble RGD, less force was needed to detach them from the surface (Puech et al., 2005). However, neither on the flat surface nor on topographies cell adhesion cannot be fully abolished with RGD peptide treatments (Puech et al., 2005; Lamers et al., 2012), and in fact, Sawyer et al. (2005) reported that RGD, alone, is not sufficient to induce a full cell attachment and spreading. This can explain why we observe cell attachment and formation of stress fibers on hMSCs, despite the RGD treatment in hMSCs on both flat surfaces and microtopographies. However, the spatial organization and phenotype of stress fibers drastically change on both flat surfaces and microtopographies. Specially on microtopographies, cells appeared to be more rounded and actin fibers are more clustered. Therefore, we speculate that these are accumulations of cortical actin, which could also explain the lack of focal adhesions on microtopographies as cortical actin is often linked with cadherin-based cell–cell adhesion, rather than cell–surface adhesion (Engl et al., 2014; Chalut and Paluch, 2016). However, considering the current literature on the adhesion strength of cells on topographies (56) and RGD treatment (Puech et al., 2005), we speculate that our RGD data could suggest that topographies can mimic the RGD effect and lead to the formation of smaller focal adhesions yet this still has to be proven by assessing the adhesion strength *via* atomic force microscopy or other tools.

## Conclusion

In this article, we show that cell attachment adapts to the properties of the surface on which the cells grow. The spatial distribution of topographic features is translated into an adjusted distribution of focal adhesions, degree of maturation, and the formation of stress fibers. Our data are following the tensegrity model, which states that cells coordinate their internal stress on the actin cytoskeleton with the mechanical properties of their environment. Our data could indicate that cells on topographic surfaces have lower tension than cells on a flat surface, yet this still must be further investigated by measuring traction forces or *via* phospho-myosin light chain 2 staining, which is a marker of cytoskeletal tension. We further demonstrate that our data are consistent with the role of actin-related signal transduction pathways in the differentiation of MSCs and tenocytes under the influence of topographies and the relationship we found between cell shape and function.

## Data Availability

The raw data supporting the conclusion of this article will be made available by the authors, without undue reservation.

## Supplementary Material

Supplementary Video S1 Tenocytes cultured on the flat surface and stained with SiR-actin (purple), a live actin staining, to illustrate actin cytoskeleton and stress fibers. Images were taken every 10 min using a Leica DMI8 microscope for 40 hours and merged with Fiji.

Supplementary Video S2 Tenocytes cultured on the tendon imprint surface and stained with SiR-actin (purple), a live actin staining, to illustrate actin cytoskeleton and stress fibers. Images were taken every 10 min using a Leica DMI8 microscope for 40 h and merged with Fiji.
